# Validation of Artificial Intelligence in the Classification of Adolescent Idiopathic Scoliosis and the Compairment to Clinical Manual Handling

**DOI:** 10.1111/os.14144

**Published:** 2024-07-03

**Authors:** Lu Tingsheng, Luo Chunshan, Yao Shudan, Pu Xingwei, Chen Qiling, Yang Minglu, Chen Lu, Wang Lihang

**Affiliations:** ^1^ Department of Spine Surgery Beijing Jishuitan Hospital Guizhou Hospital Guiyang China

**Keywords:** Artificial Intelligence, Cobb Angle, Deep Learning, Lenke Classification, Scoliosis, Spine

## Abstract

**Objective:**

The accurate measurement of Cobb angles is crucial for the effective clinical management of patients with adolescent idiopathic scoliosis (AIS). The Lenke classification system plays a pivotal role in determining the appropriate fusion levels for treatment planning. However, the presence of interobserver variability and time‐intensive procedures presents challenges for clinicians. The purpose of this study is to compare the measurement accuracy of our developed artificial intelligence measurement system for Cobb angles and Lenke classification in AIS patients with manual measurements to validate its feasibility.

**Methods:**

An artificial intelligence (AI) system measured the Cobb angle of AIS patients using convolutional neural networks, which identified the vertebral boundaries and sequences, recognized the upper and lower end vertebras, and estimated the Cobb angles of the proximal thoracic, main thoracic, and thoracolumbar/lumbar curves sequentially. Accordingly, the Lenke classifications of scoliosis were divided by oscillogram and defined by the AI system. Furthermore, a man–machine comparison (n = 300) was conducted for senior spine surgeons (n = 2), junior spine surgeons (n = 2), and the AI system for the image measurements of proximal thoracic (PT), main thoracic (MT), thoracolumbar/lumbar (TL/L), thoracic sagittal profile T5–T12, bending views PT, bending views MT, bending views TL/L, the Lenke classification system, the lumbar modifier, and sagittal thoracic alignment.

**Results:**

In the AI system, the calculation time for each patient's data was 0.2 s, while the measurement time for each surgeon was 23.6 min. The AI system showed high accuracy in the recognition of the Lenke classification and had high reliability compared to senior doctors (ICC 0.962).

**Conclusion:**

The AI system has high reliability for the Lenke classification and is a potential auxiliary tool for spinal surgeons.

## Graphical abstract

It is well known that artificial intelligence (AI) has advantages in image recognition.  In this study, we developed a smart AI system that possessed multiple functions in the diagnosis of AIS patients, including proximal thoracic (PT), main thoracic (MT), and thoracolumbar/lumbar (TL/L) curves, thoracic sagittal profile T5–T12, bending views PT, bending views MT, bending views TL/L, the Lenke classification system, the lumbar modifier, and sagittal thoracic alignment. This not only provided an accurate Lenke classification strategy but also avoided duplications. The AI system showed high accuracy in the recognition of the Lenke classification and had high reliability compared to senior doctors (ICC 0.962). This article is the first to study the diagnosis of AIS patients based on AI, and satisfactory accuracy has been achieved in the calculation. Secondly, AI systems have shown high efficiency.

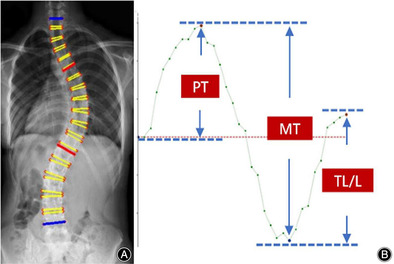

## Introduction

Adolescent idiopathic scoliosis (AIS) is among the most common spinal deformities and affects millions of children and adolescents worldwide.^1^ The Cobb's method was used to calculate the spine angle in 1948 and became the gold standard in the estimation of scoliosis in patients.^2^ The magnitude of the Cobb angle is associated with the diagnosis and decision‐making in watchful waiting, bracing and surgery, and even the selection of fusion segments for AIS patients.^3^ To date, there are two problems with applying the Cobb's method in clinical practice. First, interobserver variance is common, ranging from 4° to 8°. This is due to the difference in the identification of upper/lower vertebral endplates by personal cognition. Second, the time of 15 to 20 min per patient is unavoidable, which is required to draw the line and its vertical extension line and measuring angle. Interestingly, new emerging technologies, such as the end vertebra tilt angle method and smartphone‐aided measurement, have simplified the measuring tools used to draw endplate lines to measure Cobb angles electronically.^4^, ^5^, ^6^ The advancement of technology has also made mobile phones a medical tool. Additionally, with the development of image processing technology and artificial intelligence (AI),^7^, ^8^, ^9^ a fully automated analysis of spine shape has been proposed^10^, ^11^ using deep learning models with vertebral end plate centers labeled.^8^ Although these methods work for the standing coronal view, lateral and side‐bending views have not yet been available.^11^ These criteria have not met the clinical demands in the identification of the cephalad border and caudad border and classification of scoliosis, which limits clinical practice. In other words, the cephalad border and caudad border in their respective ranges could not be identified automatically. As such, in this study, we have developed an automatic AIS measurement system, which could automatically determine the curves by distinguishing the cephalad border and caudad border vertebra and measure the Cobb angle, based on convolutional neural networks. It could classify the objects according to Lenke classification. Eventually, the same group of patients were measured with the system and compared with the measurement data by senior spinal surgeons to illustrate its efficiency and reliability.

The accurate measurement of Cobb angles is crucial for the effective clinical management of patients with AIS. The Lenke classification system plays a pivotal role in determining the appropriate fusion levels for treatment planning. However, the presence of interobserver variability and time‐intensive procedures presents challenges for clinicians. In this study, we propose a fully automated method for measuring Cobb angles in AIS patients using convolutional neural networks. Our approach introduces the concept of oscillography to assist in identifying structural bends, which are essential for accurate angle measurement, classification of spinal curvature, and subsequent treatment decision‐making. This innovative methodology distinguishes our system from others in terms of its precision and advancement. The novelty of our approach lies in its ability to recognize structural bends, which are critical factors in the field of spinal specialization. The study aims to achieve three objectives: (i) to validate the feasibility of our developed AI measurement system for Cobb angles in AIS patients by comparing its measurement accuracy with manual measurements; (ii) to validate the reliability of our AI measurement system for Lenke classification by comparing its accuracy with manual classification; and (iii) to demonstrate the efficiency of the AI system and alleviate the workload of clinical practitioners by comparing the time required for both methods.

## Materials and Methods

### Data Harvest

This retrospective study was approved by the Ethics Committee of Guizhou Orthopedic Hospital in July 2021 (20210703). The requirement for written informed consent was waived because this was a retrospective study and presented minimal risk of harm to subjects. X‐ray images of the total spine coronal, sagittal, right‐ and left side‐bending positions from 530 patients were obtained, and 30 congenital scoliosis, neuromuscular disorders, and syndromic disorders were excluded. The 500 images were desensitized and randomly allocated to a training set (*n* = 300) and a validation set (*n* = 200). The training set was used to train automatic measurement systems. The validation set was used to compare the accuracy of trained automatic measurement systems with manual measurements. The diagnosis of AIS is determined by the imaging characteristics, natural history, and clinical presentation.

### Construction of the Automatic Measurement System

**FIGURE 1:**
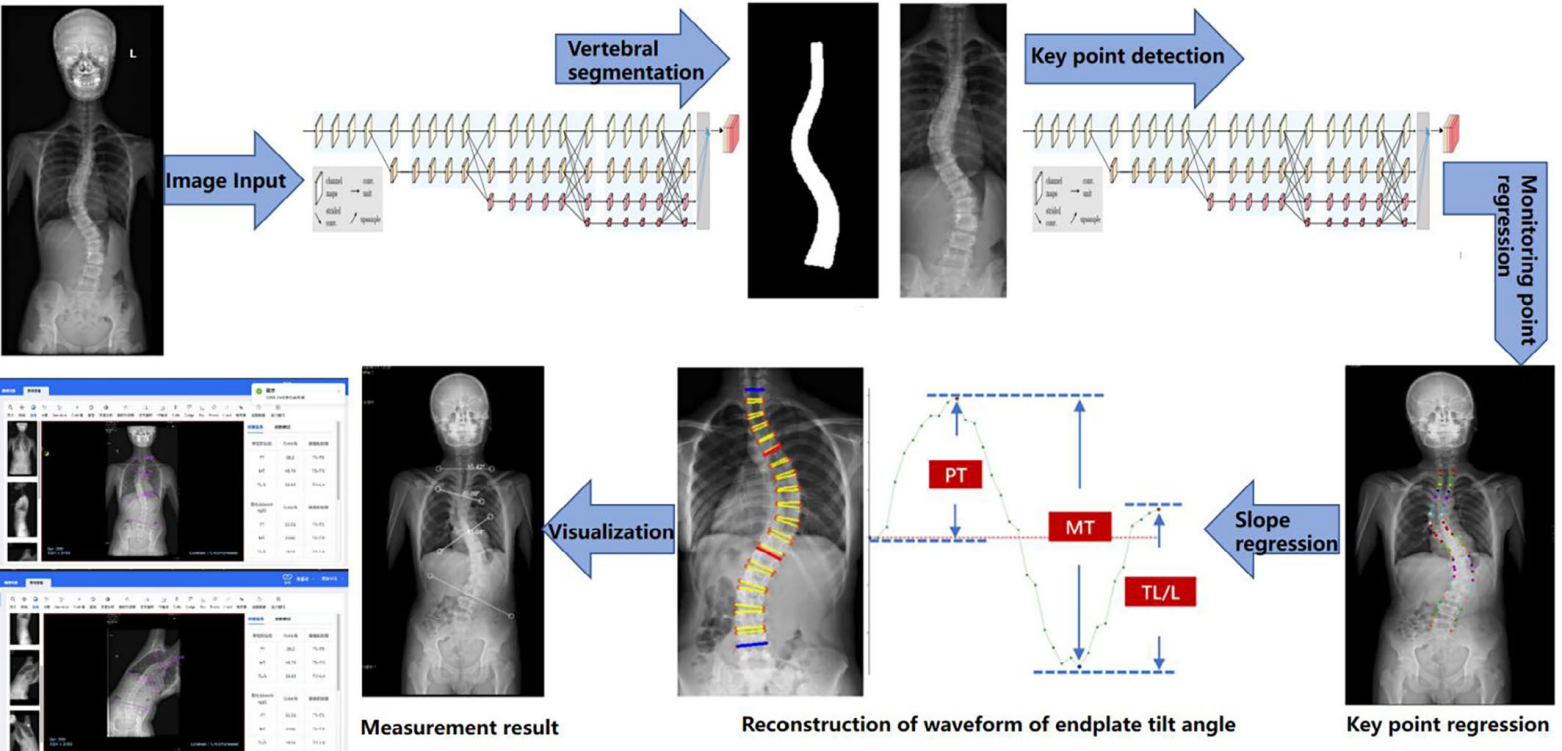
Logic diagram and process of classification algorithm.

**FIGURE 2:**
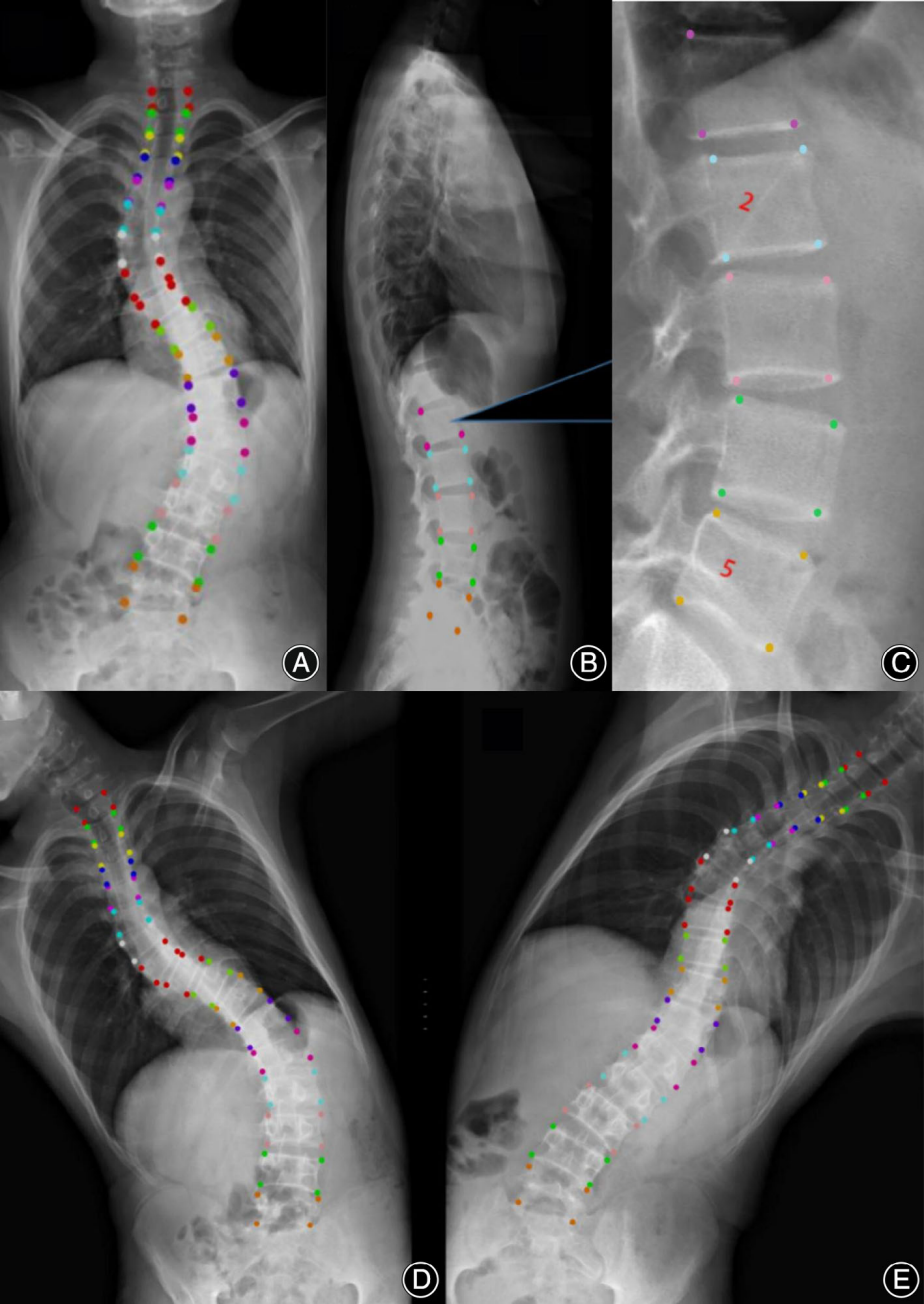
Draw points on four vertices of each vertebra for deep learning. The coronal image of whole spine boundary vertices of the vertebra from T1 to L5 were labeled manually (A); sagittal image (B) and its amplification (C). Right and left bending views (D, E).

**FIGURE 3:**
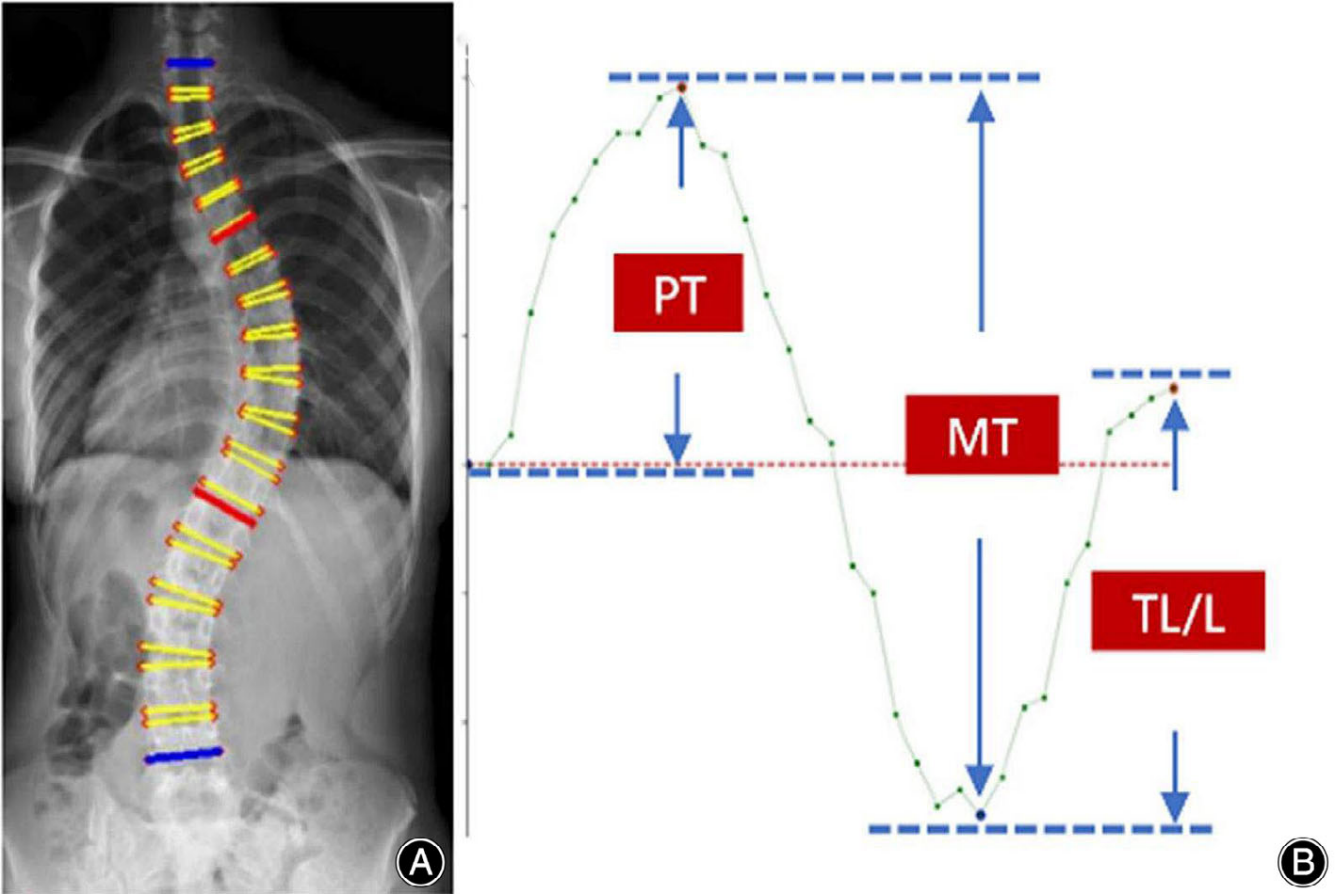
Oscillogram of the endplate slope distribution. The labeled images were used to structure the artificial intelligence endplate slope distribution (A). The oscillogram obtained from the endplate slope distribution (B). The Cobb angles of the proximal thoracic (PT), main thoracic (MT), and thoracolumbar/lumbar (TL/L) curves were identified according to the oscillogram.

**FIGURE 4:**
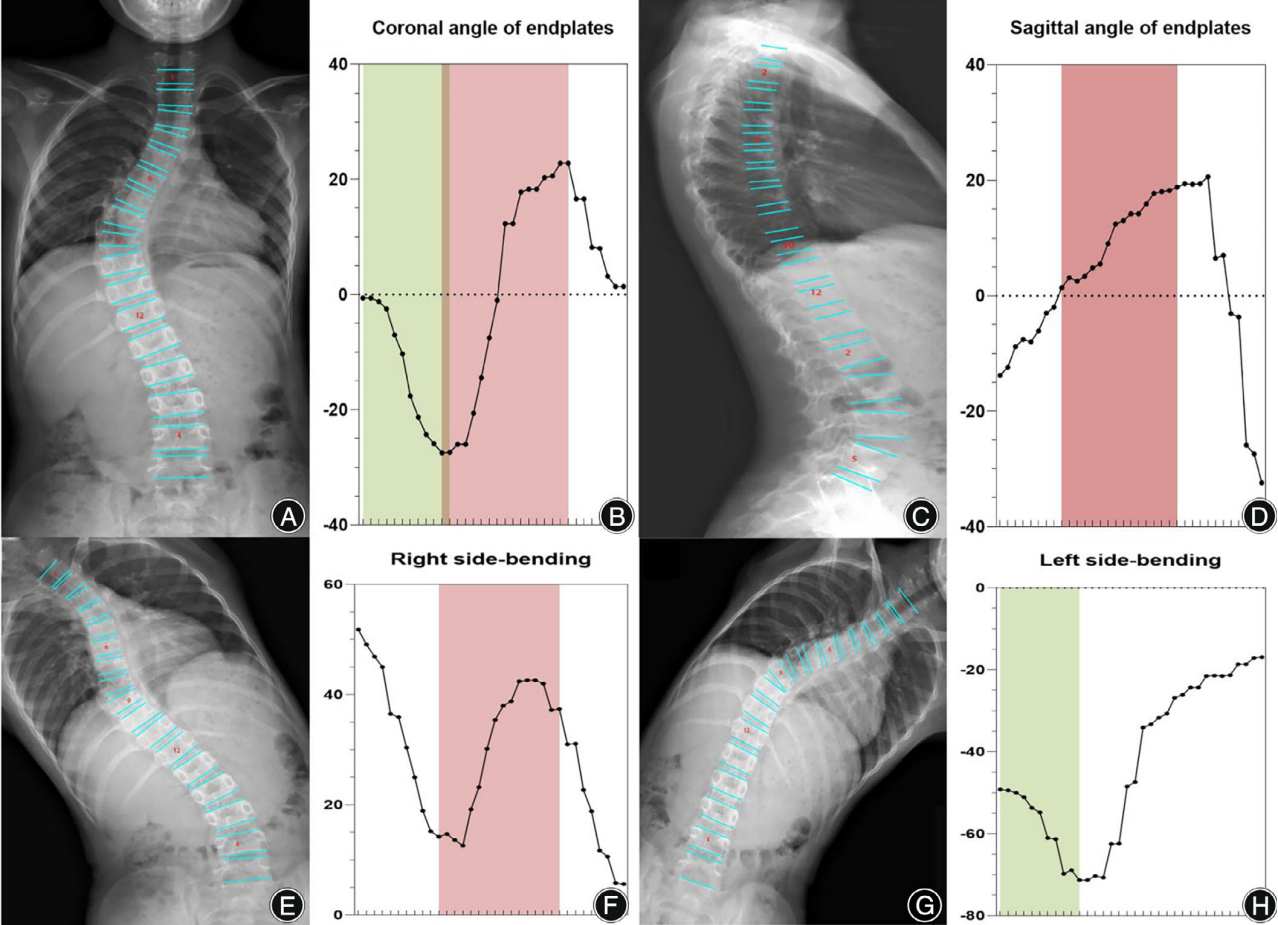
Oscillograms of Lenke 1 of adolescent idiopathic scoliosis. Coronal Lenke 1 classification image (A). The oscillogram is displayed by sinuoid, and the green part represents PT (B). The PT was less than 25° in the bending view (G, H). The Cobb angle of the main curve was calculated by the difference in the value between the trough and peak in the oscillogram (red) (B). Thoracic sagittal profile T5–T12 (C) in the oscillogram (red) (D).

**FIGURE 5:**
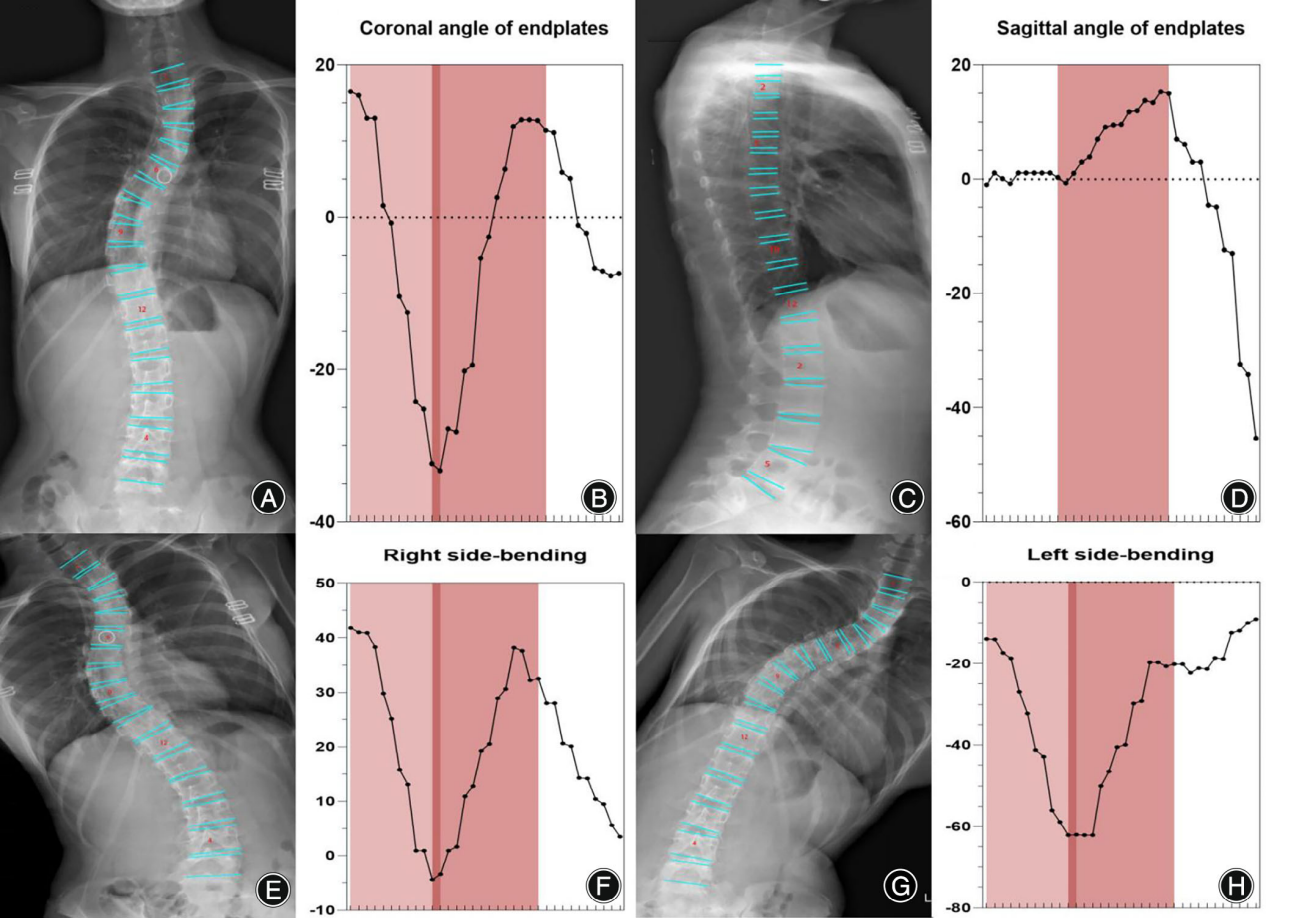
Oscillograms of Lenke 2 of adolescent idiopathic scoliosis. The coronal view of the Lenke 2 classification image (A). The Cobb angle of the structural proximal thoracic (PT) curve was calculated by the difference in the values between the left peak and the trough in the oscillogram, and the main curve was calculated by the trough and right peak (B). The red part represents structural PT and main thoracic (MT) (E, F, G, H). Thoracic sagittal profile T5–T12 (C) in the oscillogram (red) (D).

**FIGURE 6:**
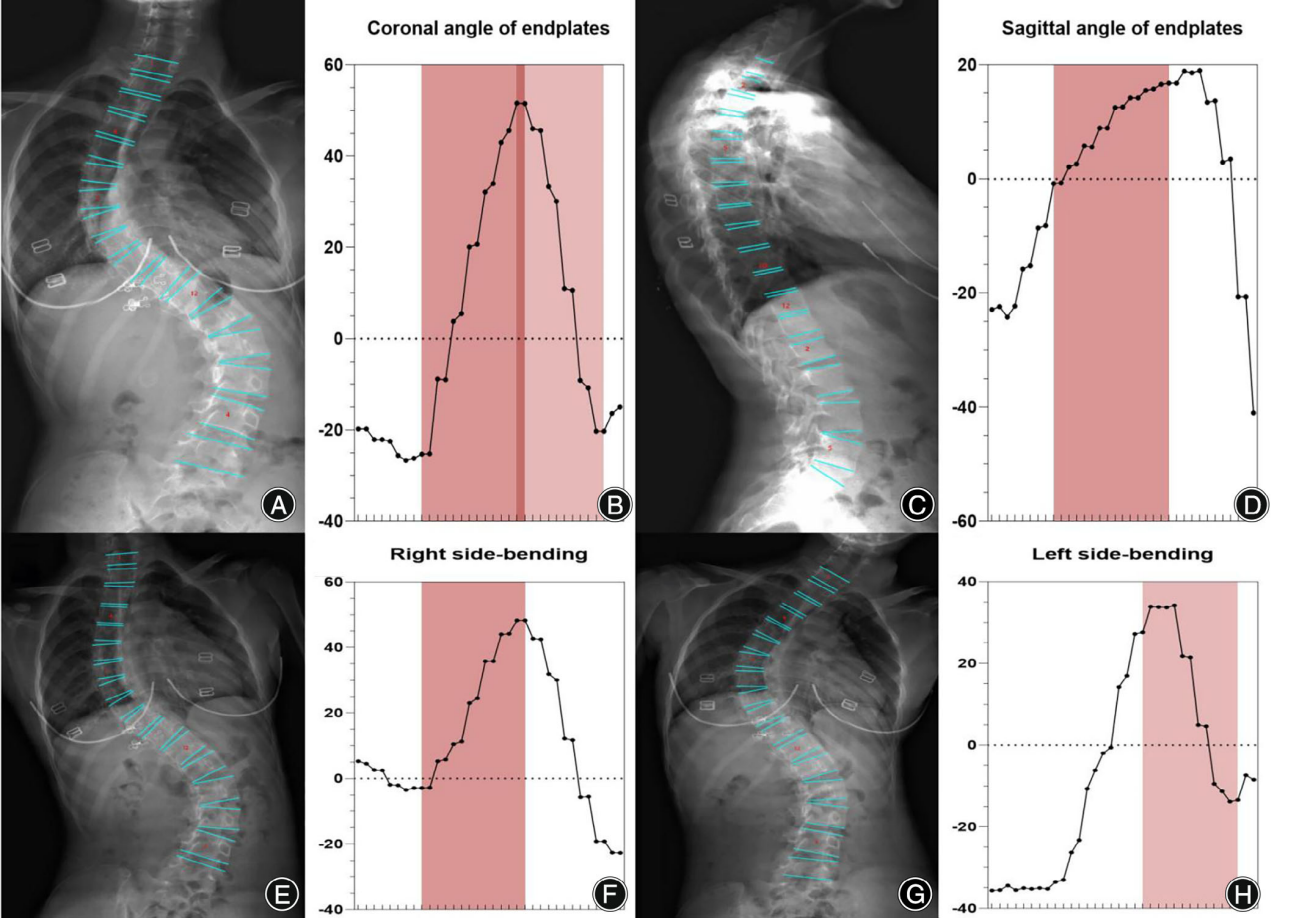
Oscillograms of Lenke 3 of adolescent idiopathic scoliosis. The coronal view of the Lenke 3 classification image (A). The Cobb angle of the structural main thoracic (MT) curve was calculated by the difference in the values between the left trough and the peak in the oscillogram, and the TL/L curve was calculated using the peak and right trough. The tail section of the oscillogram indicates that thoracolumbar/lumbar (TL/L) was not a main bend, and the difference between the peak and trough is less than that of the main curve (B). The red part represents structural MT and TL/L (E, F, G, H). Thoracic sagittal profile T5–T12 (C) in the oscillogram (red) (D).

**FIGURE 7:**
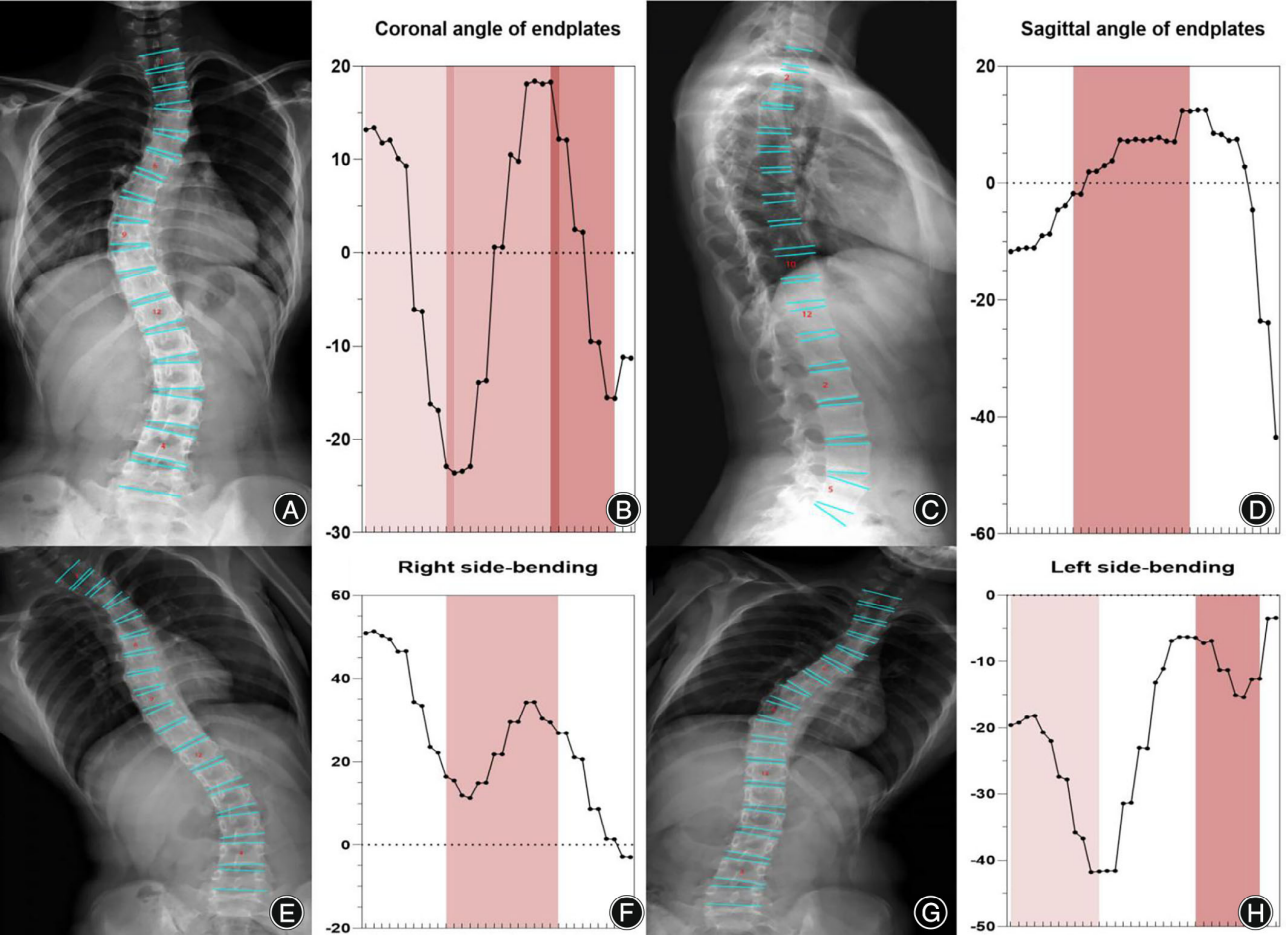
Oscillograms of Lenke 4 of adolescent idiopathic scoliosis. The coronal view of the Lenke 4 classification image (A). The oscillogram of Lenke 4 displayed a sharp broken line, and the value differences between one peak and one trough represent proximal thoracic (PT), main thoracic (MT), and thoracolumbar/lumbar (TL/L), respectively (B). The red part represents structural PT, MT, and TL/L (E, F, G, H). Thoracic sagittal profile T5–T12 (C) in the oscillogram (red) (D).

**FIGURE 8:**
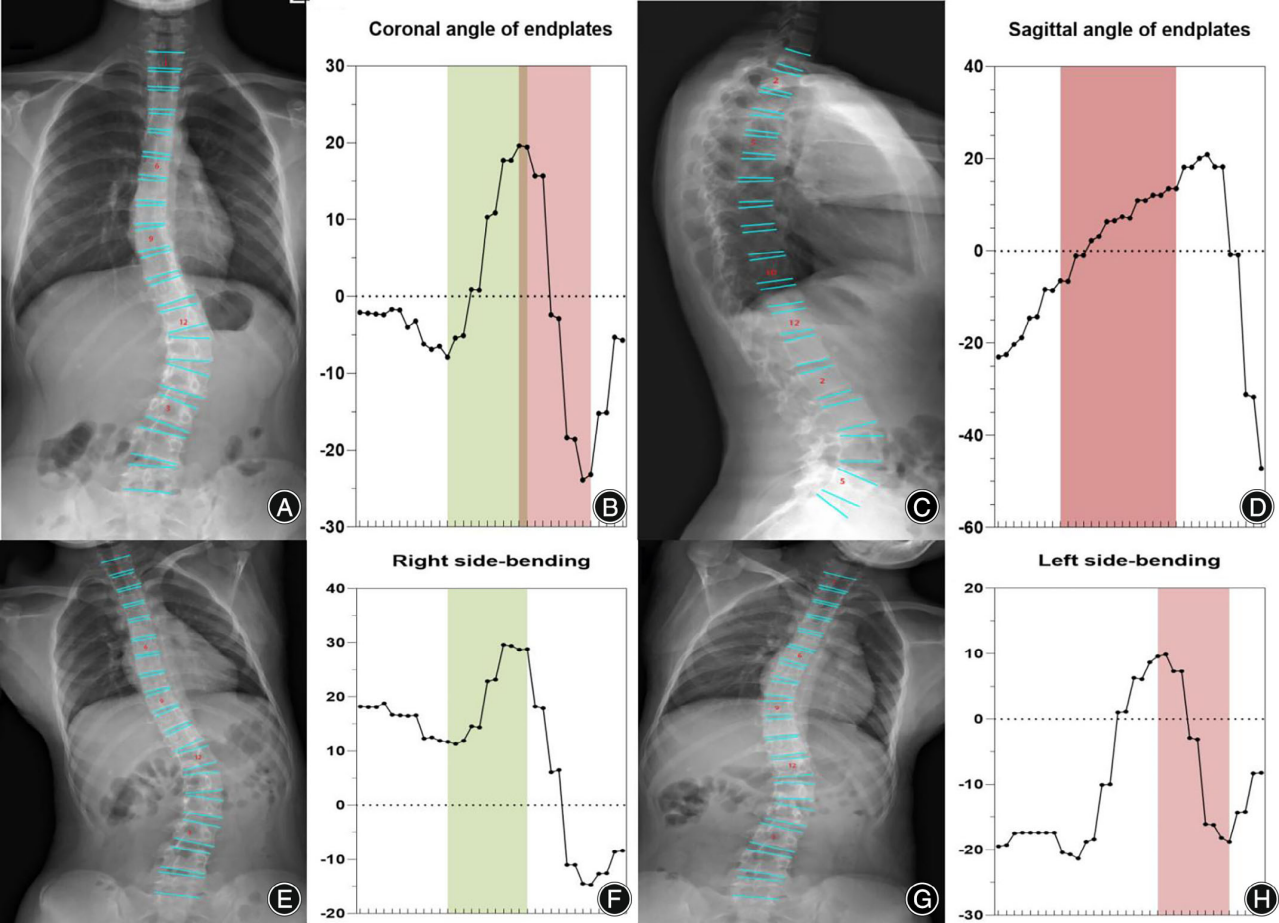
Oscillograms of Lenke 5 of adolescent idiopathic scoliosis. The coronal view of the Lenke 5 classification image (A). The oscillogram of the Lenke 5 curve was characterized by one peak and trough in the posterior segments that showed over 25° in the coronal view (red) (B) and less than 25° in the right bending view (green) (E, F). Thoracic sagittal profile T5–T12 (C) in the oscillogram (red) (D).

**FIGURE 9:**
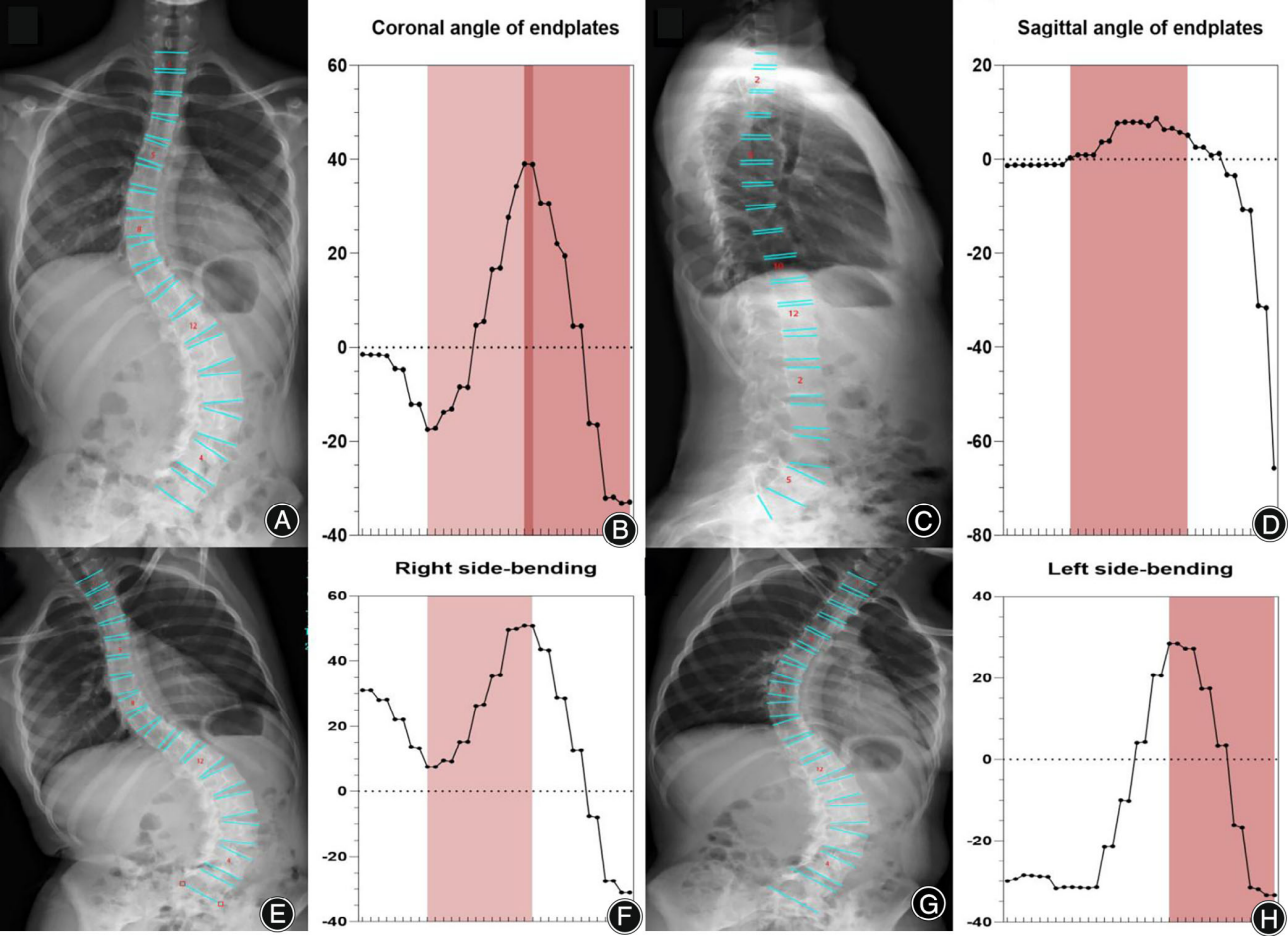
Oscillograms of Lenke 6 of adolescent idiopathic scoliosis. The coronal view of the Lenke 6 classification image (A). The Cobb angle of the structural main thoracic (MT) curve was calculated by the difference in the values between the left trough and peak in the oscillogram, and the thoracolumbar/lumbar (TL/L) curve was calculated by the peak and right trough. The tail section of the oscillogram represents TL/L as the main bend, in which the difference between the peak and trough is more than the main curve (B). The red part represents structural MT and TL/L (E, F, G, H). Thoracic sagittal profile T5–T12 (C) in the oscillogram (red) (D).

Using three common AI deep convolutional networks, namely Residual Network (ResNet), DenseNet, and Inception Network, machine measurements and deep training of feature points in spinal curvature images were performed. Based on the ResNet CNN structure, ResNet is easy to optimize, and its internal skip connection structure can alleviate the gradient vanishing problem caused by the increase in neural network depth. Therefore, this paper first constructs a CNN model based on ResNet's skip connections, which consists of eight convolutional layers, four max‐pooling layers, and one fully connected layer. Among them, the convolutional kernel size is 3 × 3, with a total of 64 convolutional kernels, and the activation function is Rectified Linear Unit (ReLU), with a max‐pooling size of 2 × 2. Batch normalization (BN) operation is added after all convolutional layers except the first two. Based on the DenseNet CNN structure, DenseNet has strong generalization ability, benefiting from its internal dense block structure, which can alleviate the overfitting problem during network training. It consists of one convolutional layer, four dense blocks, three transition layers, an average pooling layer, and a fully connected layer. The convolutional kernel size is 3 × 3, with a total of 64 convolutional kernels. Each dense block consists of a different number of dense layers, with the numbers being 3, 6, 12, and 8 for the four dense blocks, respectively. Each dense layer consists of two convolutional layers with kernel sizes of 1 × 1 and 3 × 3 and kernel numbers of 128 and 32, respectively. The transition layer is located between two adjacent dense blocks and changes the channel size to match the feature map size output by the previous dense block with the input of the subsequent dense block. The transition layer consists of one convolutional layer and an average pooling layer. The convolutional kernel size is 1 × 1, with 128 convolutions, and the average pooling size is 2 × 2. Based on the Inception Network CNN structure, the inception module uses multiple small convolutional kernels instead of large convolutional kernels, thereby increasing the use of parameters and accelerating the network's calculation speed. The network structure consists of three identical inception modules, one convolutional layer, and one fully connected layer. Each inception module consists of two different convolutional layers, one max‐pooling layer, and “four branches.” The kernel sizes of the two convolutional layers are 3 × 3, with convolutional kernel numbers of 16 and 32, respectively. The max‐pooling layer follows, with a max‐pooling size of 2 × 2. Three of the four branches are convolutional layers with kernel sizes of 3 × 3, 5 × 5, and 1 × 1, respectively. The remaining branch is an average pooling layer with a pooling size of 2 × 2. Since the operation of 1 × 1 convolutional kernels can limit the number of channels and reduce computational costs, additional convolutional layers with kernel sizes of 1 × 1 are added before convolutional layers with kernel sizes of 5 × 5 and 3 × 3 and after the average pooling layer (Figure 1). Finally, Different color labeling points were used to distinguish four vertices of each vertebra of the first thoracic to fifth lumbar vertebrae in the training set (Figure 2). The total labeled X‐ray images were used for deep learning algorithm construction and training. The learning process includes vertebral segmentation, vertebral key point detection, and Cobb angle calculation. The endplate slope distribution is shown by the oscillogram, where the peak/trough is the terminal vertebra, and the distance of the peak/trough horizontal line is the integrated Cobb angle (Figure 3). The oscillogram was plotted by the tilt angle of 34 endplates (T1–L5), and the different Lenke‐type curves represented unique characteristics in oscillograms (Figures 4, 5, 6, 7, 8, 9). The results of the human–computer interaction interface were obtained from the oscillogram.

### Oscillograms of Different Lenke Types of Adolescent Idiopathic Scoliosis

Neural network algorithms are used to classify images according to Lenke classification and visualize them using an oscillogram. The oscillogram of the Lenke 1 curve was displayed by a sinuoid that gradually falls to the bottom in the initial segment. This is a nonstructural PT and less than 25° in the bending view. The Cobb angle of the main curve was calculated by the difference in the value between the trough and peak in the oscillogram (Figure 4). The oscillogram of the Lenke 2 curve showed one sharp trough in the middle and one peak on each side, or *vice versa*. The Cobb angle of the structural PT curve was calculated by the difference in the values between the left peak and the trough in the oscillogram, and the main curve was calculated by the trough and right peak (Figure 5). The tail section of the oscillogram represents thoracolumbar/lumber curves, the difference between the peak and trough is less than the main curve, so is not a main bend (Figure 6). The oscillogram of Lenke 4 displays a sharp broken line, and the value differences between one peak and one trough represent PT, MT, and TL/L, respectively (Figure 7). The oscillogram of the Lenke 5 curve was characterized by one peak and trough in the posterior segments that showed over 25° in the coronal view and less than 25° in the right bending view. This defines the curve as structural TL/L (Figure 8). Compared to Lenke 3, Lenke 6 showed similar characteristics in the curve type and oscillogram and had a higher difference in values between the peak and trough in the latter (Figure 9). All oscillograms meet the requirements that the difference between the peak and the trough of their bending views is greater than 25°; otherwise, they cannot be counted as structural curves. The calculation method of lateral T5–T12 is different. Only the difference in the T5–T12 segment is taken as the representation of kyphosis on the oscillogram. It can be used as the basis for the correction classification of thoracic kyphosis: less than 10° is −, more than 40° is +, and the middle is N.

### Measurement Methods

The AI was tested and compared with the manual method to evaluate its accuracy. Four radiographs (total spine coronal, sagittal, and right‐ and left side‐bending views; Philips DigitalDiagnost C90, mains voltage 380 V/400 V; 50/60 Hz, three‐phase 480 V; 60 Hz, three‐phase, nominal power 65 kW or 80 kW, X‐ray tube assembly maximum voltage 150 kV) of AIS patients (n = 300) were randomly selected as a set for senior (n = 2) and junior spine surgeons (n = 2) to manually measure each twice with Surgimap (version 2.3.2), reliable software for spinal measurement.^12^, ^13^ With the AI measurement system's import of the radiographs, the measurements of each curve and the upper and lower vertebral locations were output automatically. The measurement indicators are the Cobb angle of PT, MT, TL/L, thoracic sagittal profile T5–T12, right and left bending views PT, bending views MT, bending views TL/L, the Lenke classification system, lumbar modifier, and sagittal thoracic alignment.

### Assessments of Image Quality

The image resolution is greater than 72 ppi, the image size is larger than 600 × 600, and the image color depth is greater than 8 bits. To determine the influence of image quality on the accuracy of key point identification, image quality of the major curve with mean absolute error (MAEs) exceeding 5° was assessed by pairing images with the same sex and age from the remaining images with MAE ≤ 5°. The quality of the images was evaluated on six dimensions, including bone/soft tissue contrast, bone sharpness, visibility of the processus spinosus, delignation of the intervertebral spaces, assessment of the spinal curve, and assessment of the Risser grade. One attending and one fellowship‐trained spine surgeon independently assessed the 50 images. Differences were resolved by consensus with the participation of a senior spine surgeon.

### Statistical Analysis

Statistical analysis was performed with SPSS software (version 25.0, IBM). Data were compared between two groups with the use of *t*‐tests for continuous variables and χ^2^‐tests for categorical variables. Intraobserver and interobserver reliabilities for manual evaluators were estimated by calculating the intraclass correlation coefficient (ICC) with the corresponding 95% confidence interval (CI).^14^ The consistency of the Cobb angle derived by automatic and manual methods was assessed using the MAE and ICC.

## Results

### Demographic Data

The 500 included patients were aged 8–24 years (13.55 ± 3.52 years), including 86 men, and 414 women. Of these, 200 patients were included in a validation set, with 17 males and 183 females: PT 0.0–59.9 (19.71 ± 10.35), MT 7.7–85.9 (36.53 ± 13.34), TL/L 1.3–71.9 (28.43 ± 11.71), and lateral T5–T12 0.7–59.9 (18.13 ± 7.65). The number of Lenke 1–6 cases was 77, 20, 19, 5, 70, and 9.

### Measuring Time between Artificial Intelligence and Manual Methods

The AI system measures PT, MT, TL/L, sagittal thoracic alignment, and right/left bending views of PT, MT, and TL/L, which provides Lenke classifications to guide clinical practice. The AI measurement system took no more than 200 ms to detect and calculate the above 10 parameters of each patient. Altogether, it took 30 min to measure all 300 patients and upload them to the AI system. In contrast, the measurement time for surgeons was 23.6 min per patient. It took approximately 3600 min for senior spine surgeons to measure 300 patients using the manual methods. Two measurements were made, which took approximately 7100 min, to guarantee intraobserver reliability.

### Consistency between Artificial Intelligence and Manual Methods

To validate the clinical feasibility of AI system, the accuracy of the measurements was compared among the AI system and the junior and senior spine surgeons. For PT, the paired comparison was conducted for the AI system versus senior spine surgeons, the AI system versus junior spine surgeons, and senior versus junior spine surgeons, with ICC 0.855 (*p* < 0.05), 0.809 (*p* < 0.05), and 0.863 (*p* < 0.05), respectively, and MAE 6.03 ± 7.36 (*p* < 0.05), 5.53 ± 5.66 (*p* < 0.05), and 4.64 ± 4.61 (*p* < 0.05), respectively. In MT, MAE was 4.27 ± 5.63 (*p* < 0.05), 7.37 ± 5.65 (*p* < 0.05), and 4.35 ± 4.83 (*p* < 0.05), respectively, and ICC was 0.906 (*p* < 0.05), 0.875 (*p* < 0.05), and 0.932 (*p* < 0.05), respectively. In TL/L, MAE was 5.68 ± 5.31 (*p* < 0.05), 5.56 ± 7.59 (*p* < 0.05), and 3.67 ± 4.63 (*p* < 0.05), respectively, and ICC was 0.743 (*p* < 0.05), 0.795 (*p* < 0.05), and 0.912 (*p* < 0.05), respectively. In sagittal thoracic T5–T12, MAE was 5.96 ± 10.15 (*p* < 0.05), 6.82 ± 6.06 (*p* < 0.05), and 4.07 ± 3.34 (*p* < 0.05), respectively, and ICC was 0.913 (*p* < 0.05), 0.901 (*p* < 0.05), and 0.965 (*p* < 0.05), respectively. The Lenke classification ICC was 0.962 (*p* < 0.05), 0.931 (*p* < 0.05), and 0.944 (*p* < 0.05). All results are shown in Tables 1 and 2.

**TABLE 1:** Mean absolute error of the curved Cobb angle of the artificial intelligence and manual methods.

Mean absolute error (mean ± standard deviation)	AI/senior	AI/junior	Senior/junior
Proximal thoracic (degree)	6.03 ± 7.36	5.53 ± 5.66	4.64 ± 4.61
Main thoracic (degree)	4.27 ± 5.63	7.37 ± 5.65	4.35 ± 4.83
Thoracolumbar/lumbar (degree)	5.68 ± 5.31	5.56 ± 7.59	3.67 ± 4.63
Thoracic sagittal profile T5–T12 (degree)	5.96 ± 10.15	6.82 ± 6.06	4.07 ± 3.34
Bending views proximal thoracic (degree)	5.43 ± 8.56	5.51 ± 7.44	4.55 ± 4.67
Bending views main thoracic (degree)	4.83 ± 7.56	6.06 ± 7.83	4.68 ± 4.62
Bending views thoracolumbar/lumbar (degree)	6.65 ± 7.61	6.05 ± 9.10	3.42 ± 4.03

**TABLE 2:** Intraclass correlation coefficients of artificial intelligence and manual methods.

ICC (95% CI)	AI/senior	AI/junior	Senior/junior
Lenke classification system	0.962 (0.921, 0.987)	0.931 (0.891, 0.964)	0.944 (0.894, 0.963)
Lumbar modifier	‐	‐	0.874 (0.763, 0.893)
Thoracic sagittal profile T5–T12	0.913 (0.811, 0.954)	0.901 (0.754, 0.937)	0.965 (0.924, 0.983)
Proximal thoracic	0.855 (0.812, 0.888)	0.809 (0.579, 0.897)	0.863 (0.713, 0.923)
Main thoracic	0.906 (0.807, 0.947)	0.875 (0.517, 0.948)	0.932 (0.892, 0.955)
Thoracolumbar/lumbar	0.743 (0.649, 0.810)	0.795 (0.726, 0.846)	0.912 (0.885, 0.933)
Coronal image of whole spine	0.772 (0.635, 0.850)	0.762 (0.612, 0.846)	0.785 (0.653, 0.859)
Sagittal thoracic alignment	0.588 (0.658, 0.804)	0.604 (0.508, 0.685)	0.845 (0.800, 0.880)
Bending views proximal thoracic	0.760 (0.694, 0.813)	0.719 (0.634, 0.785)	0.857 (0.771, 0.906)
Bending views main thoracic	0.813 (0.757, 0.857)	0.832 (0.784, 0.870)	0.925 (0.902, 0.943)
Bending views thoracolumbar/lumbar	0.551 (0.431, 0.650)	0.624 (0.519, 0.708)	0.885 (0.850, 0.911)

### Assessments of Image Quality

To identify the image quality influence on the assessments, 50 images with MAEs over 5° and the equivalent images under 5° were compared. Decreased overall scores (16.22 *vs*. 17.15; *P* = 0.006), bone/soft tissue contrast (2.12 *vs*. 3.09; *P* = 0.021), and bone sharpness (2.73 *vs*. 3.16; *P* = 0.025) were found in the images with MAEs over 5°. There were no significant differences in the visibility of the processus spinosus.

## Discussion

In this study, we proposed fully automated measurement of the Cobb angle in AIS patients using convolutional neural networks. Convolutional neural networks identified the boundary vertices of vertebral bodies using the encoder–decoder framework and provided efficient spinal curvature measurements *via* oscillograms automatically. The Cobb angles of PT, MT, and TL/L curves, sagittal kyphosis, right‐ and left side‐bending views and the Lenke classification, the correction classification of the thoracic kyphosis, and the lumbar modifier were measured simultaneously within 200 ms. This AI measurement took 30 min for 300 patients. In comparison, it took a total of 7100 min and 15 working days for a senior spine surgeon to measure 300 images. As such, the AI system has advantages in the efficiency of calculation and comprehensive assessments.

### The Oscillogram Established Based on the Slope of the Endplates is the Key to Lenke Classification

The clinical management of AIS patients was close to the major curves of the Cobb angle,^3^ which is one of the most important radiological parameters in scoliosis. Initially, the fuzzy Hough transform was proposed to find line structures in vertebral edge images, and high agreement between automatic and manual measurements was achieved (ICC > 0.95).^7^ Subsequently, Wu *et al*. proposed a fully automated spinal curvature assessment using MVC‐Net and obtained an MAE of 4.04°.^8^ Recently, an automated method was presented based on deep learning approaches by extracting anatomical parameters from biplanar radiographs of the spine. The standard error of the Cobb angle in scoliosis was as high as 9.9°.^9^ A recent study reported a spline construct from vertebral centroids, which obtained superior reliability compared to the traditional Cobb method.^15^ Given the progression of curving and rotation, wedging vertebral bodies emerge accordingly, which causes nonparallelization of the upper and lower endplates.^16^ In our study, the four boundary vertices of the vertebral body were implemented based on automatic images rather than the median line assessment of the vertebral body. First, 68 vertebral boundary vertices from 12 thoracic and 5 lumbar vertebrae were labeled manually. Second, 200 X‐ray images from AIS patients were labeled to train the convolutional neural networks. Third, the vertebral boundary point and vertebral sequence of the imported images were automatically recognized using the trained networks. The AI system determined the upper and lower endplates of each vertebral body based on the four boundary vertices and thereby determined 34 endplate lines for the 17 vertebrae. The AI system automatically measured the horizontal inclination angle of the 34 endplate lines and plotted the oscillogram. The peaks and troughs of the oscillogram represented the vertebral endplate with the greatest inclination angle. In addition, this method measured PT, MT, and TL/L at one time within 200 ms. The oscillogram procedure seemed to be more related to the principle of the Cobb angle in characterizing the severity of the curvature.

### Artificial Intelligence Requires Larger‐Scale Data Training to Improve the Reliability of Oscillograms

On coronal PT and MT, the system was more consistent with the manual measurement (ICC 0.855, 0.809, 0.906, and 0.875). Usually, the AI system selects the vertex of each bend based on the vertex of the oscillograph. If the vertex of the oscillograph was not a sharp turn, the AI selection appeared as a mistake and caused the vertebral selection deviation. This problem can be solved by increasing the number of machine learning samples. On sagittal thoracic alignment, the AI system had low consistency with manual measurements (ICC 0.588 and 0.604) due to limited recognition, including thoracic, scapular, and humeral occlusion. To our knowledge, 3D recognition technology might be useful and has the potential to solve the defects of lateral tablet recognition. The novelty of our approach lies in the recognition of structural bends, which are of great importance in the field of spinal specialization. We have introduced the concept of oscillography to aid in identifying structural bends, which are crucial for angle measurement, classification of spinal curvature, and subsequent treatment selection. This sets us apart from other measurement systems in terms of differentiation and advancement.

### Oscillograms Are Helpful in Identifying Structural Curves in Scoliosis

The AI system had advantages in the recognition of the Lenke classification and had the highest consistency with senior doctors (ICC 0.962, 0.931). This is based on the principle of an oscillogram, even exceeding the judgment of junior spine surgeons, which is very gratifying. As shown in the results section, identifying the upper and lower vertebra with the oscillogram model method was an ideal way to handle and determine complex human decisions. Oscillograms can convert complex scoliosis images into computer‐recognized language after computer processing and then output the corresponding Lenke classification and angle to guide clinical decision‐making. Lenke classification is recognized to be important in the clinical management of AIS patients, which eventually determines the selection of fusion levels. If the fusion levels are excessive, the quality of life in AIS patients is seriously reduced. Otherwise, the scoliosis is residual. To date, there have been no reports or products on AI imaging classification and surgical recommendations for scoliosis. For the AI system, surgical recommendations were based on the output of the results, which had been verified by a senior spine surgeon. In practical applications, the frequent issues were focused on the upper and lower vertebrae of structural curves between the AI system and spine surgeons. In our comparison, it was found that the AI for the upper vertebra of the PT was more inclined to the first thoracic, and spine surgeons preferred the second thoracic. The AI for the lower vertebra of the PT was more inclined to the superior vertebra than spine surgeons. The difference in the selection of upper and lower vertebrae of structural curves was the root cause of the low consistency. Interestingly, MT and TL/L were consistent.

## Limitations and Strengths

The shortcomings of this study were that the imaging data of each medical institution might differ slightly, including but not limited to image clarity, projection angle, and stitching. While these differences might seem negligible to our physicians’ eyes, they create significant gaps for computer recognition. Additionally, the training dataset used to train our model was collected from a single center. This is also a problem that we need to address further, and we hope to expand our training dataset to include data from multiple centers to train our model for broader applicability.

The primary focus of this manuscript is to alleviate the burden on surgeons from simple repetitive tasks using AI methods while enhancing measurement accuracy and continuously improving the feasibility of this approach.

## Conclusion

The AI system has high reliability for the Lenke classification and could be a potential auxiliary tool for spinal surgeons.

## Conflict of Interest Statement

The authors declare no competing financial or non‐financial interests. No potential conflict of interest was reported by the authors.

## Author Contribution

Lu Tingsheng and Luo Chunshan contributed equally to this work and are responsible for experimental design, implementation, and data analysis. Yao Shudan was responsible for experimental implementation. Pu Xingwei and Chen Qiling were responsible for evaluation and system testing. Yang Minglu and Chen Lu were responsible for data collection. Wang Lihang was responsible for experimental implementation and project supervision. Conflicts of interest. The authors declare no conflicts of interest.

## Data Availability

The datasets generated during and/or analyzed during the current study are available from the corresponding author on reasonable request.
